# Decline in Carbon Monoxide Transfer Coefficient in Chronic Obstructive Pulmonary Disease

**DOI:** 10.3390/jcm9051512

**Published:** 2020-05-18

**Authors:** Yeon Wook Kim, Chang-Hoon Lee, Hun-Gyu Hwang, Yu-Il Kim, Deog-Kyeom Kim, Yeon-Mok Oh, Sang Haak Lee, Ki Uk Kim, Sang-Do Lee

**Affiliations:** 1 Division of Pulmonary and Critical Care Medicine, Department of Internal Medicine, Seoul National University Bundang Hospital, Seongnam 13620, Korea; kimyw@snu.ac.kr; 2 Division of Pulmonary and Critical Care Medicine, Department of Internal Medicine, Seoul National University College of Medicine, Seoul 03080, Korea; kimdkmd@snu.ac.kr; 3 Department of Medicine, Soonchunhyang University Gumi’s Hospital, Asan 39371, Korea; hwangpark@hanmail.net; 4 Department of Internal Medicine, Chonnam National University Hospital, Gwangju 61469, Korea; kyionly@chonnam.ac.kr; 5 Seoul Metropolitan Government-Seoul National University Boramae Medical Center (SMG-SNU Boramae Medical Center), SNU, Seoul 07061, Korea; 6 Division of Pulmonary and Critical Care Medicine, Asan Medical Center, University of Ulsan College of Medicine, Seoul 05505, Korea; ymoh55@amc.seoul.kr (Y.-M.O.); sdlee@amc.seoul.kr (S.-D.L.); 7 Department of Internal Medicine, Eunpyeong St. Mary’s Hospital, College of Medicine, The Catholic University of Korea, Seoul 03312, Korea; agmante@gmail.com; 8 Department of Internal Medicine, Pusan National University School of Medicine, Busan 49241, Korea; uk303@hanmail.net

**Keywords:** chronic obstructive pulmonary disease, carbon monoxide transfer coefficient, exacerbation, lung function decline

## Abstract

Background: Although a reduced carbon monoxide transfer coefficient (Kco) is an important feature in chronic obstructive pulmonary disease (COPD), how it changes over time and its relationship with other clinical outcomes remain unclear. This study evaluated longitudinal changes in Kco and their relationship with other clinical outcomes. Methods: We evaluated patients with COPD from the Korean Obstructive Lung Disease cohort, followed up for up to ten years. Random coefficient models were used to assess the annual change in Kco over time. Participants were categorized into tertiles according to Kco decline rate. Baseline characteristics and outcomes, including changes in FEV1 and emphysema index, incidence of exacerbations, and mortality, were compared between categories. Results: A decline in Kco was observed in 92.9% of the 211 enrolled participants with COPD. Those with the most rapid decline (tertile 1) had a lower FEV1/FVC% (tertile 1: 43.8% ± 9.7%, tertile 2: 46.4% ± 10.5%, tertile 3: 49.2% ± 10.4%, *p* = 0.008) and a higher emphysema index at baseline (27.7 ± 14.8, 22.4 ± 16.1, 18.1 ± 14.5, respectively, *p* = 0.001). Tertile 3 showed a lower decline rate in FEV1 (16.3 vs. 27.1 mL/yr, *p* = 0.017) and a lower incidence of exacerbations (incidence rate ratio = 0.66, 95% CI = 0.44–0.99) than tertile 1. There were no differences in the change in emphysema index and mortality between categories. Conclusion: Most patients with COPD experienced Kco decline over time, which was greater in patients with more severe airflow limitation and emphysema. Decline in Kco was associated with an accelerated decline in FEV1 and more frequent exacerbations; hence, this should be considered as an important outcome measure in further studies.

## 1. Introduction

Chronic obstructive pulmonary disease (COPD) is characterized by persistent airflow limitation and related chronic respiratory symptoms due to airway and/or parenchymal abnormalities. COPD is primarily caused by air pollution, including smoking, and influenced by host factors [1,2]. Many patients with COPD experience acute exacerbations with disease progression, leading to mortality and morbidity, creating an economic and social burden worldwide [3,4]. A decline in forced expiratory volume in one second (FEV1) is a well-known indicator of disease progression in COPD and has been used as an important outcome parameter in many COPD studies [5,6,7]. However, it is known that FEV1 alone does not adequately reflect disease severity such as parenchymal destruction, decreased exercise performance, and clinical symptoms [8,9,10]. 

The carbon monoxide transfer coefficient (Kco) is regarded as an index to assess the efficiency of alveolar transfer of carbon monoxide by measuring the pulmonary gas exchange across the alveolar–capillary membrane [11]. Kco could decline not only with parenchymal destruction but also with small airway diseases and microvascular destruction [11], which are important pathological changes seen in COPD but not necessarily linked to the degree of airway obstruction [12,13]. A decreased Kco is also associated with increased pulmonary venous pressure and cardiac problems that affect the prognosis of COPD [11,14,15]. Thus, we could postulate that Kco is also related to COPD prognosis. However, to date, limited data are available on the association between Kco and COPD outcomes [7]. Moreover, changes in Kco over time and the relationship between these changes and COPD outcomes, such as a decline in FEV1, acute exacerbations, and mortality, have never been reported.

Therefore, we conducted a multicentre prospective cohort study among patients with COPD to examine the variability of changes in Kco over time during follow-up and to investigate the relationship between these changes and COPD outcomes including the annual decline rate of FEV1, rate of exacerbations, and all-cause mortality [16].

## 2. Materials and Methods 

### 2.1. Study Design and Participants

Data collected on the Korean Obstructive Lung Disease (KOLD) cohort were used in this study. We prospectively recruited patients diagnosed with obstructive lung disease from pulmonary clinics of 14 referral hospitals in Korea between June 2005 and October 2012, and followed them up for up to ten years. Details of this cohort are reported previously [16]. The KOLD cohort initially excluded patients who had respiratory diseases other than obstructive lung disease, and patients with comorbidities that can interfere with the study results (e.g., malignancies, congestive heart failure, chronic renal failure, uncontrolled hypertension). To include only COPD patients and evaluate the change in Kco, patients who met the following inclusion criteria were enrolled in the present study: (1) were older than 40 years; (2) had post-bronchodilator FEV1/forced vital capacity (FVC) < 0.7; (3) were current or ex-smokers with a smoking history of over 10 pack-years; and (4) had more than two annual measures of Kco. Baseline information of participants included demographic characteristics and smoking status, symptom scores from the St. George’s respiratory questionnaire (SGRQ) and the modified Medical Research Council (mMRC) dyspnea-scale, and history of acute exacerbations in the year preceding enrolment. In addition to regular follow-ups at 3-month intervals, reports were collected when patients experienced acute exacerbations or all-cause mortality throughout the follow-up period. Acute exacerbation was defined as any event that required an unplanned visit to an emergency room or clinic with or without admission due to the aggravation of respiratory symptoms. Written informed consent was provided by all included patients at baseline enrolment into the cohort. The study was conducted according to the principles of the Declaration of Helsinki. This study design was approved by the ethics committee of the Seoul National University Hospital Institutional Review Board (IRB no. 1611-013-804). As the KOLD study was initiated in 2005, the protocol was not registered in an international clinical trial registry.

### 2.2. Lung Function Measurements

Pulmonary function tests were performed according to the American Thoracic Society guidelines using Vmax 22 (Sensor Medics, Yorba Linda, CA, USA) and PFDX (Medgraphics, St. Paul, MN, USA) [17]. Post-bronchodilator FEV1 and FVC, total lung capacity (TLC), residual volume (RV), and Kco were measured at baseline and at each annual visit. Post-bronchodilator spirometry values were measured 15 min after administering 400 µg of salbutamol. Bronchodilator reversibility was defined as an increase in FEV1 that was 12% above the baseline value and at least 200 mL after administration [18]. Lung volumes, TLC and RV, were measured using body plethysmography with V6200 (CareFusion, San Diego, CA, USA), PFDX, or Vmax 22 [17]. Values for diffusing capacity (DLco) and predicted alveolar volume (VA) were measured by assessing the single-breath carbon monoxide uptake (Vmax 22 or PFDX). Measures of DLco were adjusted for hemoglobin concentrations using the equation provided by American Thoracic Society guidelines [19]. Kco values were calculated by dividing measures of hemoglobin-adjusted DLco (mmol/min/mmHg) by VA (L) [7,20]. 

### 2.3. Chest CT Measures

Volumetric computed tomography (CT) scans were taken upon enrolment, after one year, and subsequently at intervals of three years. CT scans were taken at full inspiration and expiration using three 16-multidetector CT scanners produced by different manufacturers (Somatom Sensation 16; Siemens Medical Systems, Bonn, Germany; GE Lightspeed Ultra; General Electric Healthcare, Milwaukee, WI, USA; and Philips Brilliance 16, Philips Medical Systems, Best, The Netherlands). Images of the whole lung were extracted automatically, and the attenuation coefficient of each pixel was calculated. Emphysema index (volume fraction of the lung ≤  −950 Hounsfield Units (HU), air trapping index (mean lung density at full expiration/mean lung density at full inspiration), and percentage wall area (wall area percentage of two segmental bronchi; RB1 and LB1 + 2) were measured for quantitative assessment. 

### 2.4. Statistical Analysis

Random coefficient models with random slopes and intercepts were used to estimate the Best Linear Unbiased Prediction (BLUP) of annual changes in Kco (mmol/min/mmHg/L per year) for each patient and to establish the effect of patient characteristics on the annual change rate of Kco [7,21]. To investigate the potential relationship between patient characteristics and changes in Kco and the relationship between these changes and other clinical outcomes, participants were categorized into tertiles based on the degree of annual change in Kco (tertile 1: those with the most rapid decline, tertile 3: those with the slowest decline). Annual changes in FEV1, emphysema index, and SGRQ score were calculated using random coefficient models with random slopes and intercepts for each patient who had two or more longitudinal measures of post-bronchodilator spirometry, CT exams, and SGRQ score, respectively. 

Characteristics between groups were compared using the *t*-test and one-way analysis of variance, as appropriate. A binomial negative regression analysis was performed to evaluate the relationship between each group and annual exacerbation rates in terms of incidence rate ratio (IRR), with adjustments for the following factors: age, sex, body mass index, smoking status, pack-years smoked, baseline FEV1, exacerbation history at baseline, and use of inhaled corticosteroid/long-acting β-agonists (ICS/LABA), or inhaled long-acting muscarinic antagonists (LAMA). Mortality between groups was analyzed using Cox-proportional hazards modelling, with adjustments for the same covariates listed above. The 95% confidence intervals (CIs) were calculated, and *p* < 0.05 was considered to indicate statistical significance. All analyses were performed using IBM SPSS Statistics 25.0 (IBM Corp., Armonk, NY, USA) and Stata version 14.2 (StataCorp, College Station, TX, USA). 

## 3. Results

### 3.1. Patient Characteristics and the Rate of Change in Kco

**Figure 1:**
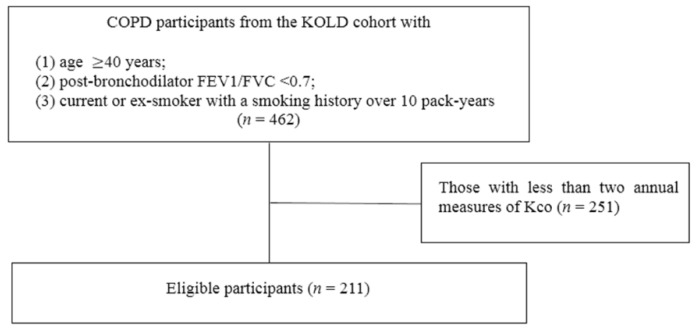
Flow diagram of the study design.

Of the 462 patients with COPD from the KOLD cohort, 211 participants were eligible for analyses (Figure 1). The mean follow-up period of the enrolled subjects was 6.1 ± 2.7 years. 

**Figure 2:**
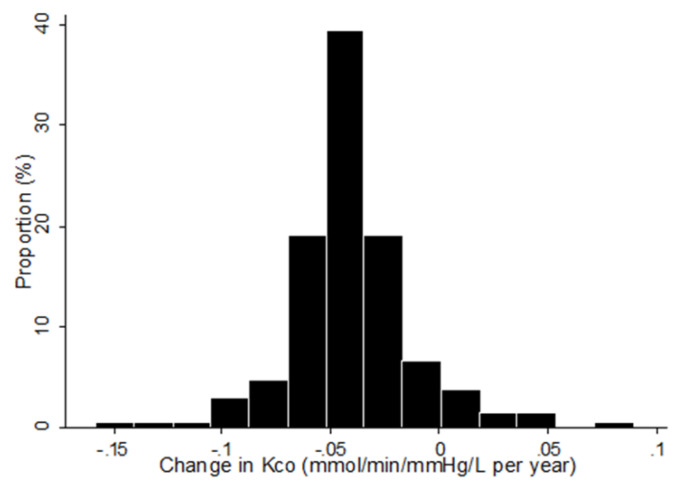
Distribution of estimated annual rates of change in Kco (*n* = 211). Footnotes: Best Linear Unbiased Prediction (BLUP) of the annual change in Kco was calculated for each patient using the random-coefficient model, and the data are shown as a histogram. Mean ± SD = −0.04 ± 0.03 (mmol/min/mmHg/L per year).

The linear mixed-effects model revealed that most patients (92.9%) experienced a decline in Kco over time. The rate of change in Kco was a decline of 0.04  ± 0.03 mmol/min/mmHg/L per year, with significant variations in the levels of change (Figure 2). At baseline, measures of Kco showed significant correlation with measures of emphysema index (*r* = −0.612, *p* < 0.001) and post-bronchodilator FEV1 in liters (*r* = 0.371, *p* < 0.001). 

Demographic data and clinical characteristics of the participants across tertiles of annual change of Kco are listed in Table 1. The mean decline rate of Kco per year was 0.07 ± 0.02 for tertile 1 (most rapid decliners), 0.04 ± 0.00 for tertile 2, and 0.01 ± 0.02 for tertile 3 (slowest decliners), respectively (*p* < 0.001). There were no significant differences in BMI, smoking status and amount, or quality of life and symptom measurements among the three groups at baseline. Spirometry results revealed that baseline FEV1/FVC was positively related to the annual decline rate of Kco, showing lower baseline FEV1/FVC (%) for rapid decliners for Kco (43.8% ± 9.7% for tertile 1, 46.4% ± 10.5% for tertile 2, 49.2% ± 10.4% for tertile 3, *p* = 0.008). Measurements of CT indices showed that patients with a higher emphysema index at baseline showed a more rapid decline in Kco over time (27.7 ± 14.8 for tertile 1, 22.4 ± 16.1 for tertile 2, 18.1 ± 14.5 for tertile 3, *p* = 0.001).

### 3.2. Comparison of Changes in FEV1, Emphysema Index, and SGRQ Score According to Changes in Kco Over Time 

**Figure 3:**
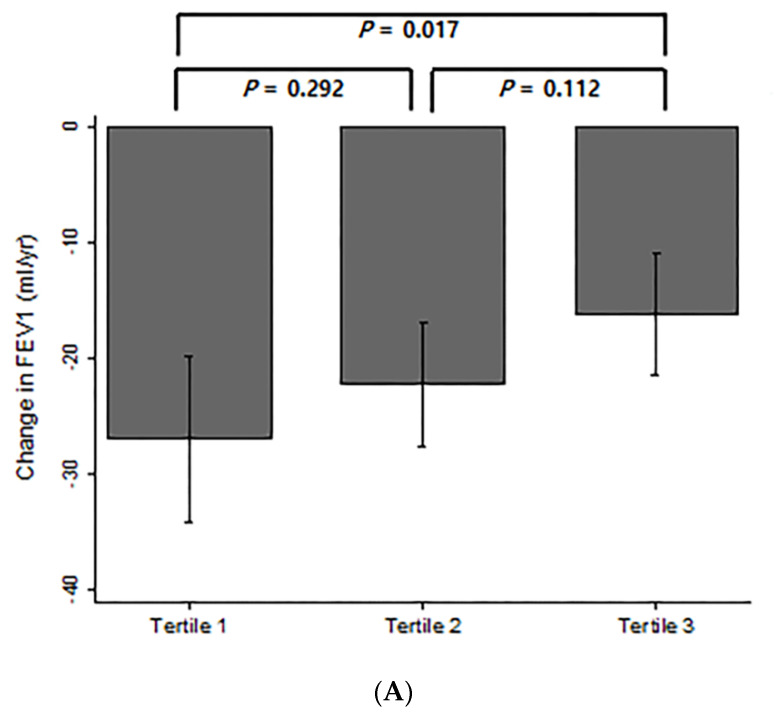

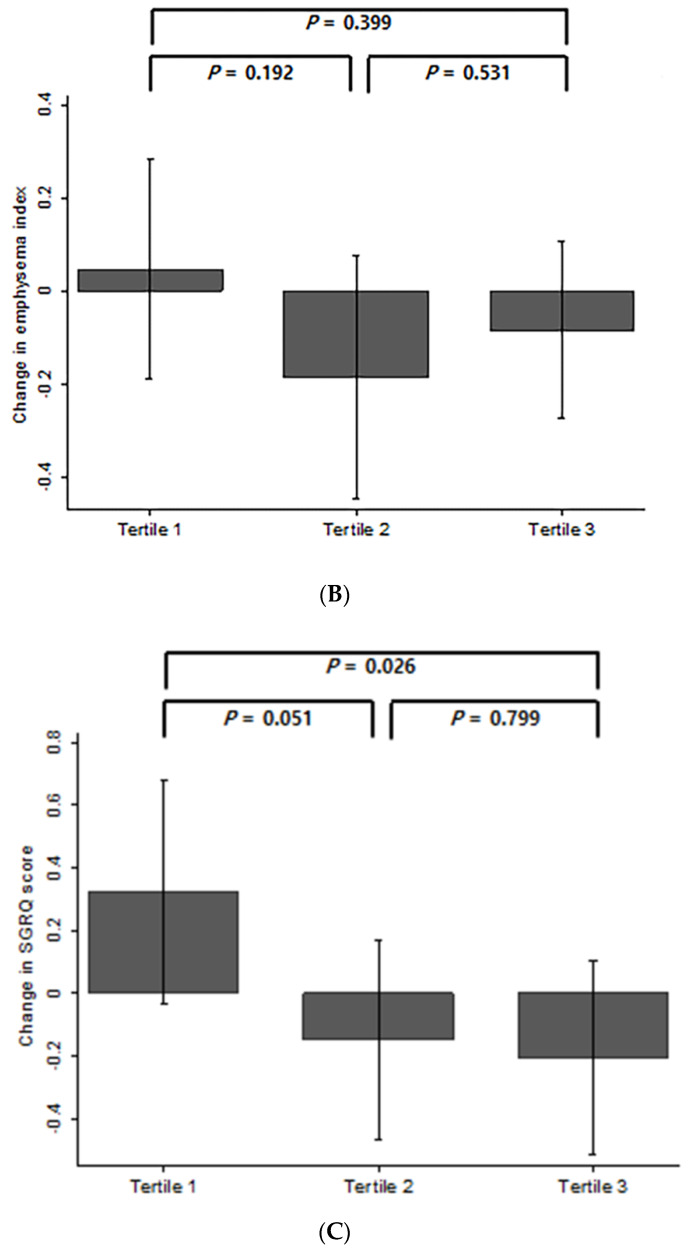
Comparison of annual rates in change of (**A**) post-bronchodilator FEV1, (**B**) emphysema index, and (**C**) SGRQ score by annual rates of decline in Kco. Comparisons of the risk of acute exacerbations and mortality in COPD according to changes in Kco over time.

All study participants had two or more annual measures of post-bronchodilator FEV1. Figure 3A shows the annual change in FEV1 over time among the three groups depending on the degree of annual decline in Kco. Compared to the group with the most rapid decline for Kco (tertile 1), those with the slowest decline (tertile 3) also showed a significantly lower decline rate in FEV1 over time (27.1 ± 30.2 mL/yr vs. 16.3 ± 21.9 mL/yr, *p* = 0.017). The changes in emphysema index among groups were compared among the 198 participants who had two or more longitudinal performance of CT exams. The annual changes in emphysema index over time in subjects, classified by the degree of annual decline in Kco, are shown in Figure 3B. The annual change in emphysema index did not significantly differ between groups. A total of 210 patients had two or more longitudinal results of SGRQ score. Figure 3C shows the annual change in SGRQ score over time among the three groups. Whereas the group with the most rapid decline for Kco (tertile 1) showed an increase of 0.32 ± 1.50 in SGRQ score, those with the slowest decline (tertile 3) showed a decrease of 0.21 ± 1.30 in SGRQ score (*p* = 0.026).

**Figure 4:**
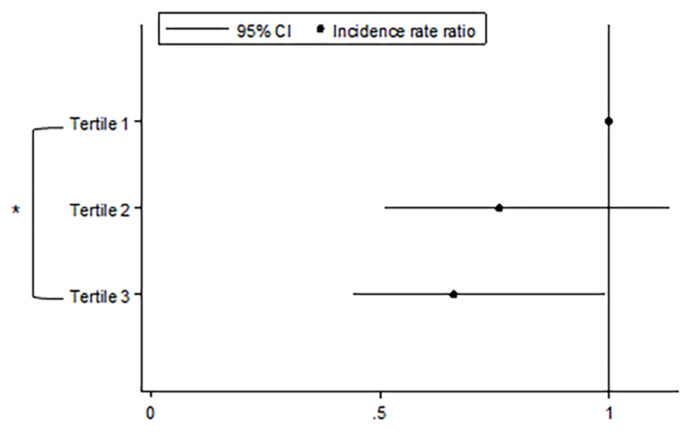
Comparison of the incidence rate of acute exacerbation according to annual rates of decline in Kco. Footnotes: All statistical analyses were adjusted for age, sex, body mass index, smoking status, pack-years smoked, baseline post-bronchodilator FEV1, exacerbation history at baseline, and use of inhaled corticosteroid/long-acting β-agonists (ICS/LABA), or inhaled long-acting muscarinic antagonists (LAMA), * *p*-value < 0.05.

**Figure 5:**
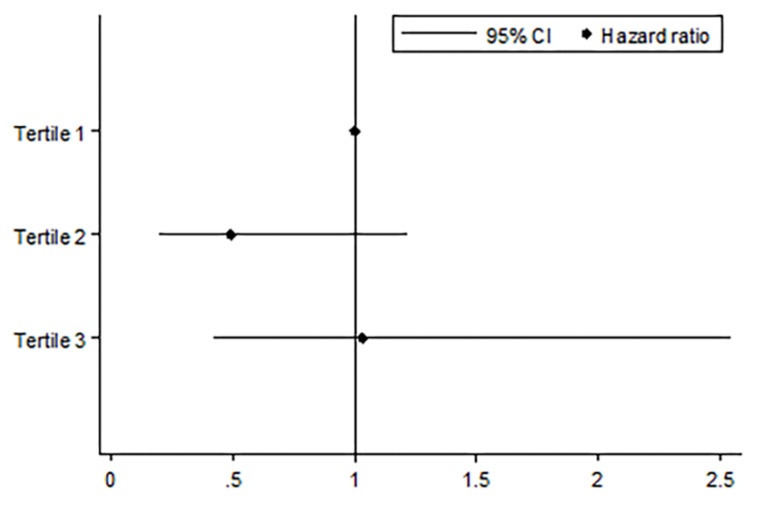
Comparison of mortality risk by annual rates of decline in Kco. Footnotes: All statistical analyses were adjusted by age, sex, body mass index, smoking status, pack-years smoked, baseline post-bronchodilator FEV1, exacerbation history at baseline, and use of inhaled corticosteroid/long-acting β-agonists (ICS/LABA), or inhaled long-acting muscarinic antagonists (LAMA).

Figure 4 shows the comparison of IRRs of acute exacerbation among tertiles classified by changes in Kco over time. Compared to patients who showed the most rapid decline in Kco over time (tertile 1), patients with the slowest decline rate in Kco (tertile 3) had a significantly lower incidence of acute exacerbation (IRR = 0.66, 95% CI = 0.44–0.99, *p* = 0.045). The trend of decreasing incidence rates from tertile 1 to tertile 3 was also significant (IRR = 0.81, 95% CI = 0.66–0.99, *p* = 0.042). Figure 5 shows the comparison of mortality risk according to groups classified by the degree of Kco decline over time. The risk of all-cause mortality did not significantly differ between groups. 

## 4. Discussion

To the best of our knowledge, this is the first study investigating changes in Kco and their association with outcomes of COPD over a long period. This study demonstrated that most patients with COPD experienced an overall decline in Kco during a mean of 6.1 years. The rate of annual change in Kco varied substantially among the patients. Patients with the highest decline rate in Kco (tertile 1) showed the lowest FEV1/FVC and the highest emphysema index at baseline. A patient with COPD who had a greater decline in Kco also showed a greater decline rate in FEV1 and a higher rate of acute exacerbations. However, we did not find the decline rate in Kco to be significantly associated with the change in emphysema index and the risk of all-cause mortality.

Traditionally, the decline in FEV1 has been widely accepted as one of the most important outcome measures reflecting disease progression in COPD [5,6]. However, there are concerns that FEV1 alone does not adequately reflect disease severity and various phenotypes [8,22,23]. Inconsistent results from previous studies evaluating the clinical significance of FEV1 in COPD support this view. For example, evidence from many prior studies shows that a low initial FEV1 is a predictor of increased risk of exacerbation and mortality [24,25,26,27,28]. In contrast, some studies revealed that a high initial FEV1 is associated with a more rapid decline in FEV1 over time [23,29]. Although COPD is currently defined on the basis of the degree of airflow limitation, the decline rate in FEV1 does not correlate well with health status and important clinical outcomes such as exacerbations and mortality [9]. Moreover, it is also known that a substantial proportion of patients with COPD do not show a decline in FEV1. In the ECLIPSE study, approximately 15% of study participants showed a positive annual change in FEV1 [5]. A study from the Hokkaido COPD cohort revealed that approximately one fourth of the patients with COPD did not experience a significant decline in FEV1 [7]. In the BODE cohort, only 18% of those enrolled revealed a significant decline in FEV1 [23]. Accordingly, recent studies have attempted to investigate changes in other important features such as the progression of emphysema or hyperinflation and COPD-related outcomes [22,30,31]. However, limited data exist on the relationship between progression of such indices and other outcomes. Similarly, there are discrepancies around the factors that are shown to predict the various clinical course of COPD [30,32]. 

Intriguingly, the results from our study showed that most (92.9%) patients with COPD experienced a decline in Kco over time, which is different from the trajectories of FEV1. The decline in Kco was associated with baseline disease severity as well as subsequent outcomes. Interestingly, patients with a lower initial FEV1/FVC and a higher emphysema index experienced a more rapid decline in Kco over time. However, baseline FEV1 was not significantly associated with the decline rate of Kco. This could be explained by the better correlation between emphysema severity and FEV1/FVC than the correlation between emphysema severity and FEV1 reported from previous studies [33,34]. Our results indicate that the decline in Kco is mainly affected by the degree of baseline parenchymal destruction and emphysema rather than the degree of airway obstruction.

On the other hand, a more rapid decline in Kco was also associated with a more rapid decline in FEV1, which is known to be an indicator of disease progression. A more rapid decline in Kco was also associated with an increase in SGRQ score, which reflects the grade of symptoms and quality of life in COPD patients, with higher scores indicating more limitations. In addition, a rapid decline in Kco was also related to a higher risk of exacerbations, which is an important outcome of COPD. These findings suggest that the decline in Kco can accurately reflect the features and prognosis of COPD. 

Measurement of Kco with the single-breath method is considered to reflect changes in functional lung volume and impairment in gas transport across the alveolar–capillary membrane. Thus, Kco indicates the degree of parenchymal destruction, reduced alveolar surface, and loss of pulmonary capillary density in patients with COPD [24]. Therefore, a reduction in Kco would reflect progression in alveolar destruction and emphysema, which are important phenotypes of COPD [11,35]. The good correlation observed between Kco and emphysema index, as quantified by CT at baseline in our study, supports the close pathophysiological relation between the two indices for reflecting disease status and is in accordance with the findings of previous studies [36,37]. However, it is known that neither measures from CT scans nor Kco are perfect predictors of emphysema severity on a pathologic basis, and thus they should be regarded as complementary measurements [38]. In our study, the relationship between the decline in Kco and changes in emphysema measured by CT was not observed. The discrepancy between the change in Kco and the change in emphysema index on CT could be explained by the fact that changes in Kco can be affected by various factors in addition to emphysema. First, a Kco decline is in part related to a gradual reduction in alveolar–capillary density along with decreased pulmonary capillary blood volume, which are the main determinants of Kco in patients with COPD [39,40,41]. Second, changes in Kco can reflect the changes that precede visible emphysema such as bronchiolitis and injury of the terminal airspace that result in dysfunction of the distal gas exchange units [42]. Third, an increase in pulmonary venous pressure such as in pulmonary edema or left heart failure, which are common comorbidities that appear in the clinical course of COPD, can also result in Kco reduction [11]. These pathophysiology-based explanations could also help readers to understand the significant association between the decline in Kco and worse prognosis in patients with COPD, as shown in our study. A decline in Kco might provide more information on the disease progression in COPD than the emphysema index. Thus, we carefully suggest that decline in Kco should be closely monitored in clinical practice and should also be considered as a useful intermediate or outcome measure in further studies on COPD. 

Our study has limitations. First, the majority of the included subjects were male, and the findings may not be generalized to female patients with COPD since the manifestations of the disease may differ by gender [43]. This biased gender distribution would be due to the marked difference in prevalence of smoking between men and women in South Korea [44]. Second, concerns regarding the possibility of the inclusion of patients with combined pulmonary fibrosis and emphysema (CPFE), which would accelerate the decline of Kco due to the interstitial lung disease portion, and asthma, in which Kco would probably not decline, can be raised about our study population. However, the inclusion of CPFE and pure asthma patients would have been minimized owing to our study design. The original KOLD cohort initially excluded patients that had respiratory diseases other than obstructive lung disease, including interstitial lung disease, upon recruitment [16]. Moreover, it is reported that the prevalence rate of CPFE among COPD patients is very low [45]. For asthma, although the original KOLD cohort did not use smoking history as an inclusion criterion to allow the inclusion of asthma patients in the whole cohort, we have set a separate inclusion criterion of positive smoking history over 10 pack-years to evaluate definite COPD patients from the original cohort with exclusion of pure asthma patients. In our study, 26 (12.3%) of the 211 participants showed a positive bronchodilator reversibility. However, it is known that not only asthma but also COPD patients can show positive bronchodilator reversibility [46]. Although we could not precisely report how many participants in our study would be classified into asthma-COPD overlap due to lack of data, considering reports from previous studies, only a small proportion of COPD patients with a positive bronchodilator reversibility are expected to be those with asthma-COPD overlap [47,48]. Third, our study did not fully evaluate all biomarkers that can be possibly associated with the decline rate in Kco. Considering that genetic determinants and circulating biomarkers of progression in COPD are an important area of research, additional studies will be needed to search for potential related biochemical predictors [49].

The main strength of our study is the well-designed prospective cohort with a stringent diagnosis of COPD including patients at all stages of severity, as well as the long observation period. KOLD is purely an observational study, and the observed changes are likely to represent disease-related changes in patients who were properly managed. In addition, strict records of demographic data and the standardized methodology used for evaluating lung function and imaging variables support the validity of our results. 

## 5. Conclusions

In conclusion, measures of Kco declined over time in most patients with COPD, and the decline was greater in patients with more severe airflow limitation and emphysema. A decline in Kco was also associated with an increased decline rate in FEV1 and more frequent exacerbation. Thus, Kco decline could be considered as an important outcome measure in further clinical studies.

## Figures and Tables

**Table 1:** Baseline characteristics of patients with COPD classified by annual rates of decline in Kco.

Characteristics	Tertile 1 (Group with the Most Rapid Decline, *n* = 71)	Tertile 2 (*n* = 70)	Tertile 3 (Group with the Slowest Decline, *n* = 70)	*p*-Value *
Annual change in Kco (mmol/min/mmHg/L per year)	−0.07 ± 0.02	−0.04 ± 0.00	−0.01 ± 0.02	<0.001
Age	66.8 ± 6.8	68.1± 6.6	64.7 ± 7.4	0.014
Men, *n* (%)	70 (98.6)	68 (97.1)	65 (92.9)	0.180
BMI, kg/m^2^	22.8 ± 2.9	22.2 ± 3.2	23.5 ± 3.3	0.079
Smoking status at baseline, *n* (%)				0.165
Current smokers	25 (35.2)	17 (24.3)	28 (40.0)	0.129
Former smokers	46 (64.8)	53 (75.7)	42 (60.0)	
Pack-years of smoking	51.4 ± 30.7	48.2 ± 28.6	48.6± 26.9	0.775
Total SGRQ score	37.7 ± 17.3	34.5 ± 17.0	34.1 ± 17.6	0.411
mMRC grade	1.9 ± 1.0	1.6 ± 1.1	1.6 ± 0.8	0.154
Exacerbation in previous year baseline, *n* (%)	13 (18.3)	19 (27.1)	13 (18.6)	0.348
Eosinophil count, cells/µL	312.8 ± 380.4	275.1 ± 192.4	313.6 ± 507.2	0.798
Hemoglobin, g/dL	14.9 ± 1.7	14.9 ± 1.1	14.9 ± 1.0	0.917
Baseline pulmonary function				
FEV1, L	1.5 ± 0.5	1.6 ± 0.5	1.6 ± 0.6	0.553
FEV1, % predicted	57.6 ± 18.2	57.9 ± 16.2	60.5 ± 19.5	0.588
FVC, L	3.5 ± 0.6	3.4 ± 0.8	3.3 ± 0.8	0.341
FEV1/FVC, %	43.8 ± 9.7	46.4 ± 10.5	49.2 ± 10.4	0.008
Bronchodilator reversibility, *n* (%)	9 (12.7)	7 (10.0)	10 (14.3)	0.738
Kco, mmol/min/mmHg/L	2.8 ± 0.9	2.9 ± 0.9	3.0 ± 1.0	0.539
RV/TLC, %	46.2 ± 13.1	46.0 ± 13.0	49.7 ± 13.8	0.188
Baseline CT indices				
CT emphysema index	27.7 ± 14.8	22.4 ± 16.1	18.1 ± 14.5	0.001
CT air-trapping index	94.6 ± 2.8	95.1 ± 3.6	94.2 ± 3.6	0.335
Percentage wall area, %	66.3 ± 4.8	67.7 ± 4.3	66.9 ± 5.0	0.207

* Statistical significance was examined among the groups using the analysis of variance. COPD: chronic obstructive pulmonary disease; Kco: carbon monoxide transfer coefficient; BMI: body mass index; SGRQ: St. George’s respiratory questionnaire; mMRC: the modified Medical Research Council; FEV1: forced expiratory volume in 1 s; FVC: forced vital capacity; RV: residual volume; TLC: total lung capacity; CT: computed tomography.
